# Volumetric Modulated Arc Therapy vs. c-IMRT for the Treatment of Upper Thoracic Esophageal Cancer

**DOI:** 10.1371/journal.pone.0121385

**Published:** 2015-03-27

**Authors:** Wu-Zhe Zhang, Tian-Tian Zhai, Jia-Yang Lu, Jian-Zhou Chen, Zhi-Jian Chen, De-Rui Li, Chuang-Zhen Chen

**Affiliations:** 1 Department of Radiation Oncology, Cancer Hospital of Shantou University Medical College, Guangdong, China; 2 Center of Clinical Oncology, The University of Hongkong-Shenzhen Hospital 1, Shenzhen, China

## Abstract

**Objective:**

To compare plans using volumetric-modulated arc therapy (VMAT) with conventional sliding window intensity-modulated radiation therapy (c-IMRT) to treat upper thoracic esophageal cancer (EC).

**Methods:**

CT datasets of 11 patients with upper thoracic EC were identified. Four plans were generated for each patient: c-IMRT with 5 fields (5F) and VMAT with a single arc (1A), two arcs (2A), or three arcs (3A). The prescribed doses were 64 Gy/32 F for the primary tumor (PTV64). The dose-volume histogram data, the number of monitoring units (MUs) and the treatment time (TT) for the different plans were compared.

**Results:**

All of the plans generated similar dose distributions for PTVs and organs at risk (OARs), except that the 2A- and 3A-VMAT plans yielded a significantly higher conformity index (CI) than the c-IMRT plan. The CI of the PTV64 was improved by increasing the number of arcs in the VMAT plans. The maximum spinal cord dose and the planning risk volume of the spinal cord dose for the two techniques were similar. The 2A- and 3A-VMAT plans yielded lower mean lung doses and heart V_50_ values than the c-IMRT. The V_20_ and V_30_ for the lungs in all of the VMAT plans were lower than those in the c-IMRT plan, at the expense of increasing V_5_, V_10_ and V_13_. The VMAT plan resulted in significant reductions in MUs and TT.

**Conclusion:**

The 2A-VMAT plan appeared to spare the lungs from moderate-dose irradiation most effectively of all plans, at the expense of increasing the low-dose irradiation volume, and also significantly reduced the number of required MUs and the TT. The CI of the PTVs and the OARs was improved by increasing the arc-number from 1 to 2; however, no significant improvement was observed using the 3A-VMAT, except for an increase in the TT.

## Introduction

Esophageal cancer (EC) is one of the most common malignant diseases in China, with a crude incidence and mortality of 19.24 and 15.39 per 100,000 residents, respectively. Treatment options for patients with non-distal metastatic thoracic esophageal carcinoma include fractionated external beam radiotherapy (EBRT) or surgery (by itself or combined with EBRT), either with or without chemotherapy. More than 60% of patients are diagnosed at locally advanced stages that cannot be completely resected. Thus, radiotherapy (RT) has become the major treatment method, especially for upper thoracic EC. However, treatment planning for RT of the upper thoracic EC is relatively challenging because these tumors are often located deep within the thorax, which is adjacent to several critical organs including the spinal cord, the lungs and the trachea. Inappropriate dosing may result in either recurrence of the disease or severe toxicity. Thus, it is important to investigate novel radiation delivery techniques to improve the dose coverage of this tumor and spare normal tissues.

Conventional intensity-modulated radiation therapy (c-IMRT) can be used to provide superior coverage of the planning target volume (PTV) compared to three-dimensional conformal radiation therapy (3DCRT)[1–3]. Many clinical studies have yielded good dosimetric results and patient treatment outcomes using IMRT. VMAT is a novel form of IMRT in which radiation treatment is delivered during gantry rotation with dynamic multi-leaf collimator (MLC) motion, variable dose rates (DR) and gantry speed modulation. Many studies have shown that VMAT can produce dosimetrically equivalent plans to IMRT for centrally located cancers, such as prostate cancer, cervical cancer and head and neck cancers[4–6].VMAT also requires a shorter treatment time (TT) and fewer monitoring units (MUs) than IMRT, thereby overcoming the primary drawbacks of IMRT. A shorter treatment time may make patients feel more comfortable and reduce motion errors during treatment delivery. Decreasing the amount of MUs used could reduce undesirable irradiation of normal tissue and minimize the risk of secondary cancer.

In a study by Benthuysen, the use of VMAT yielded equivalent tumor coverage and protection for organs at risk (OARs) and PTV as using IMRT, while reducing the required MUs and TTs in distal esophageal cancer[7]. However, none of the previously published reports, except the aforementioned study by Liyin[8], have addressed these issues for upper thoracic EC.

The objective of this pilot study was to evaluate the feasibility of using VMAT for thoracic EC and to formulate an optimal plan design for this technique by comparing tumor coverage, OAR sparing, MUs and TTs for VMAT and c-IMRT plans. The effect of arc number on the dose distribution in the VMAT plans was also analyzed.

## Materials and Methods

### Patients

CT datasets were identified for 11 patients (8 males and 3 females), who had pathologically confirmed upper thoracic esophageal squamous cell carcinomas and underwent radical radiotherapy at Shantou University Cancer Hospital in 2008. This retrospective study was approved by the Ethics Committee at Shantou University Medical College Tumor Hospital. All of the participants provide their written informed consent to participate in this study. The lengths of the tumor lesions ranged from 4.5 to 10.5 cm, with a median of 6.0 cm and a mean of 6.3 cm. The volumes of the esophageal tumors were 5.9–50.95 cm^3^, with a median of 21.0 cm^3^ and a mean of 25.1 cm^3^. The tumor characteristics are summarized in Table 1.

**Table 1:** Tumor characteristics.

No	Stage	Length	Volume of primary tumor (cm^3^)	Volume of LN (cm^3^)
1	T_3_N_0_M_0_	6.50	40.01	0.00
2	T_3_N_1_M_0_	7.50	28.31	0.00
3	T_2_N_0_M_0_	5.50	10.83	0.00
4	T_4_N_1_M_0_	5.50	34.96	2.28
5	T_4_N_1_M_0_	10.50	50.95	3.04
6	T_3_N_0_M_0_	6.00	29.26	0.00
7	T_3_N_0_M_0_	7.00	20.88	0.00
8	T_3_N_0_M_0_	4.50	11.05	0.00
9	T_2_N_0_M_0_	5.00	5.90	0.00
10	T_3_N_0_M_0_	4.50	22.13	1.31
11	T_3_N_0_M_0_	6.50	21.36	0.00

### Simulation

All of the patients were immobilized with a head and upper thoracic thermoplastic mask in a supine position with their arms alongside their bodies. All of the CT datasets were acquired using a helical CT scanner (Philips Brilliance CT Big Bore Oncology Configuration, Cleveland, OH, USA). The CT images were taken at a 5-mm thickness throughout the entire thorax and neck that extending to 10 cm beyond the borders of the tumor. The data were transferred to the Eclipse 10.0 treatment planning system (Varian Medical Systems, Palo Alto, CA, USA) according to the DICOM 3.0 protocol.

### Delineation of target volumes and organs at risk

The gross tumor volume (GTV) and clinical target volumes (CTVs) were contoured by senior physicians. The GTV, including the esophageal cancer and involved positive regional lymph nodes, was defined using CT images, barium swallow fluoroscopy and endoscopy. Two CTVs were contoured to cover potential microscopic diseases. The CTV_64_ was delineated with 5–10 mm radial and 20 mm longitudinal margins with respect to the GTV. The CTV_54_ was derived from the CTV_64_ plus the lymph node draining area in the upper mediastinum and bilateral supraclavicular region. Irrelevant bony structure and lung tissue were excluded from the CTVs. The PTV was created by adding a 5-mm margin to the relative CTV. The following OARs were considered: the lungs, the heart and the spinal cord with its planning risk volume (PRV-spinal cord, spinal cord plus a 5-mm margin).

### Planning techniques and objectives

All of the treatment plans were generated to deliver 64 Gy and 54 Gy to the PTV_64_ and PTV_54_, respectively, in 32 fractions using the Eclipse 10.0 treatment planning system (Varian Medical Systems, Palo Alto, CA, USA) with a 6-MV photon beam from a Varian Truebeam linear accelerator (Varian Medical Systems, Palo Alto, CA, USA), which was equipped with an MLC with 120 leaves (with a spatial resolution of 5 mm at the isocenter for the central 20 cm and 10 mm for the outer 20 cm). Both of the plans were optimized at a maximum DR of 600 MU/min. Continuous gantry motion, dose-rate variation, and MLC motion were approximated by optimizing individual beams at 2° gantry angle increments. The dose calculation was performed using the anisotropic analytical algorithm (AAA, version 8.6.02) using a 2.5-mm grid. The planning objective for the PTV_64_ was at least 95% of the PTV volume that received 100% of the prescribed dose, with Dmin > 60 Gy, Dmax < 70 Gy and V_105%_ < 5%. The following dose constraints were defined for the OARs: spinal cord, D_max_ < 40 Gy; PRV-Spinal cord, D_max_ < 45 Gy; heart, V_30_ < 30%; and lung, V_20_ < 25% and V_30_ < 20%. Fulfilling the objectives for the PTV was a higher priority than fulfilling those for the OARs.To meet the prescribed and the OAR-limited, planning goals, dose-volume constraints such as volume and weight were fine-tuned for all of the plans during inverse planning using the direct machine parameter optimization method. To ensure consistency of the planning techniques, all of the treatment plans were devised by physicists with over 8 years of clinical experience in IMRT, and over 3-years in VMAT planning.The planning requirement and techniques for planners were also aligned by training with standard protocols, and procedures in the department.

### c-IMRT plans

The c-IMRT plans were created using five coplanar fields (5F; gantry angles: 0°, 72°, 144°, 216° and 288°). The MLC leaf sequences of the plans were generated using a dynamic fixed field sliding window IMRT delivery technique.

### VMAT plans

Coplanar arcs with full gantry rotation and opposite rotation (clockwise or counter-clockwise) were used for each plan and were optimized simultaneously: a single arc for 1A, two arcs for 2A and three arcs for 3A (clockwise, counter-clockwise or clockwise, respectively). The field size and the collimator rotation were determined using an automatic tool from Eclipse to encompass the PTV. Each individual arc was limited to a sequence of 177 control points; therefore, two and three coplanar arcs were used to increase the modulation factor during optimization to simultaneously achieve increase the target homogeneity and reduce the involvement of the OARs.

After treatment planning was completed, the plan was normalized to cover 95% of the PTV with 100% of the prescribed dose. In the present study, the constraints and priority factors in the c-IMRT and VMAT plans were modified to optimize the results by adjusting the parameters as a function of the dose-volume histogram (DVH) data for each patient.

### Evaluation tools

The lans pwere evaluated using the DVH data. D_x_ was the specific dose that was computed for a fraction of a target or an organ volume, and V_x_ was the volume that was irradiated above a designated dose. The mean dose (D_mean_) and the maximum dose (D_max_) for the PTV were analyzed, where the CI of PTV_64_ was defined as (1)

$$CI=\frac{V_{T}{}_{,ref}}{V_{T}}\times \frac{V_{T,ref}}{V_{ref}}$$ (1)

The homogeneity index (HI) of the PTV was computed as in (2):

$$HI=\frac{D_{5\%}}{D_{95\%}}$$ (2)

(i.e.the difference between the dose covering 5% and 95% of the PTV). The DVH parameters for the OARs (the spinal cord, the PRV-spinal cord, the heart and the lungs) were calculated and compared using a *t*-test.

### Statistical analyses

The Statistical Package for Social Science (SPSS, Version 19.0, Chicago, IL, USA) was used to perform the statistical analyses. A normal distribution test showed that all of the data were normally distributed. The data for the different plans were reported as the average ± standard deviation (X±S) and compared using a paired two-tailed t-test. Differences in the data were considered to be statistically significant for *P* < 0.05.

## Results

### Target coverage, conformity and dose homogeneity

**Fig 1:**
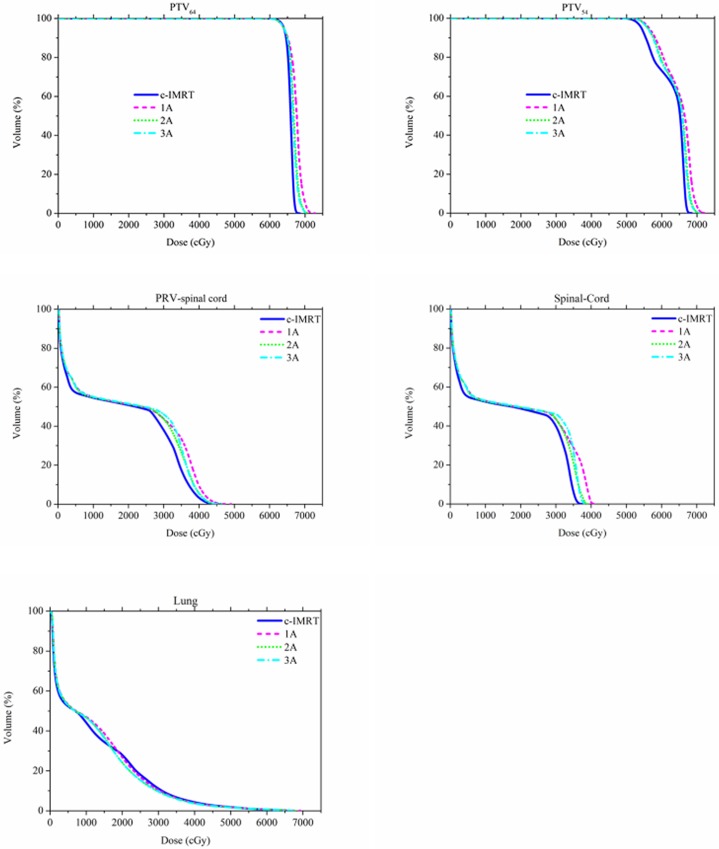
Comparison of dose-volume histograms (DVHs) for four plans.

All of the VMAT and c-IMRT plans were clinically acceptable. The VMAT and c-IMRT results are listed in Table 2. The DVH of the PTV for one patient is shown in Fig. 1. The 2A and 3A VMAT plans generated a significantly better CI for PTV_54_ than the c-IMRT plan. There was no significant difference between the 2A and 3A plans, except that the V_105_ was lower for the 3A plan (P < 0.05), thus favoring the 3A plan. Additionally, the 3A plan had a better HI for PTV_64_ than the c-IMRT plan (*P <* 0.05), whereas similar HI values were obtained for the 2A and c-IMRT plans. The HI value for the 1A plan was slightly inferior to that of the c-IMRT plan (*P <* 0.05). There were minor, but statistically significant, differences in the V_93_, V_95_ and V_105_ values of PTV_54_ between the VMAT and c-IMRT plans. The conformity and homogeneity of the VMAT plans improved as more arcs were used.

**Table 2:** Dosimetric results for planning target volume (PTV) ($\bar{X}\pm s$).

Variable	c-IMRT	1A	2A	3A	P1	P2	P3	P4	P5
PTV_64_									
D_mean_ (cGy)	6653±36	6722±28	6653±17	6631±31	0.001	0.976	0.000	0.012	0.124
V_93_ (%)	100±0.0	100±0.0	100±0.0	100±0.0	0.132	0.588	0.028	0.111	0.314
V_95_ (%)	99.9±0.1	99.8±0.1	99.9±0.0	99.9±0.1	0.017	0.566	0.040	0.105	0.821
V_105_ (%)	32.4±15.5	58.1±7.2	30.2±7.0	22.0±10.5	0.001	0.664	0.000	0.005	0.066
CI	0.86±0.03	0.85±0.02	0.88±0.02	0.89±0.02	0.133	0.046	0.001	0.089	0.008
HI	1.07±0.01	1.08±0.01	1.07±0.01	1.06±0.01	0.004	0.703	0.000	0.067	0.320
PTV_54_									
D_mean_ (cGy)	6303±114	6400±118	6325±107	6297±116	0.000	0.136	0.000	0.011	0.697
V_93_ (%)	99.9±0.1	100±0.0	100±0.0	100±0.0	0.001	0.001	0.224	0.223	0.001
V_95_ (%)	99.7±0.2	99.8±0.1	99.8±0.1	99.9±0.1	0.003	0.003	0.848	0.330	0.001
V_105_ (%)	97.9±0.7	98.8±0.6	98.6±0.7	98.3±0.8	0.003	0.013	0.010	0.030	0.102

P1: 1A vs. c-IMRT P2: 2A vs. c-IMRT P3: 2A vs.1A P4: 3A vs.2A P5: 3A vs. c-IMRT

### OARs

The dosimetric results for the spinal cord, PRV-spinal cord, heart and lungs are summarized in Table 3. The DVH for the OAR in one patient is shown in Fig. 1. The D_max_ values for the spinal cord and the PRV-spinal cord were similar for the 2A and c-IMRT plans, whereas the 3A plan showed a slight and significant trend toward better results than the c-IMRT plan (P < 0.05). Only the 1A plan achieved a higher D_max_ than the c-IMRT plan (*P <* 0.05). The VMAT plan generated a significantly lower V_20_ and V_30_ for the lungs than the c-IMRT plan (*P <* 0.05) as V_5,_ V_10_ and V_13_ were incrementally increased. Likewise, the D_mean_ of the lungs and the heart V_50_ was lower for the 2A and 3A plan than for the 1A or c-IMRT plans. No significant difference was detected between the 2A and 3A plans or the 1A and c-IMRT plans.

**Table 3:** Dosimetric results for organs at risk (OARs) ($\bar{X}\pm s$).

Variable	c-IMRT	1A	2A	3A	P1	P2	P3	P4	P5
SC-PRV									
D_max_ (cGy)	4533±160	4703±148	4501±142	4455±129	0.002	0.331	0.000	0.005	0.053
Spinal Cord									
D_max_ (cGy)	3863±82	4014±127	3841±92	3807±104	0.001	0.469	0.000	0.038	0.106
Lung									
D_mean_ (cGy)	1071±275	1059±286	1040±283	1041±283	0.337	0.013	0.001	0.602	0.016
V_5_ (%)	47.3±13.6	48.0±14.4	48.1±14.5	48.1±14.5	0.027	0.036	0.395	0.249	0.041
V_10_ (%)	39.6±11.5	41.5±12.9	41.4±13.1	41.5±13.2	0.009	0.016	0.643	0.275	0.016
V_13_ (%)	32.8±9.5	35.9±11.3	35.3±11.2	35.5±11.3	0.002	0.003	0.138	0.329	0.004
V_20_ (%)	23.7±6.8	19.9±6.5	19.1±6.1	19.1±6.0	0.001	0.000	0.007	0.890	0.000
V_30_ (%)	8.6±2.5	7.5±2.0	7.0±2.0	7.0±2.0	0.001	0.000	0.000	0.826	0.000
Heart									
D_mean_ (cGy)	480±564	472±531	480±542	480±543	0.548	0.999	0.096	0.760	0.934
V_20_ (%)	7.9±12.9	7.8±11.8	8.2±12.4	8.1±12.4	0.925	0.591	0.061	0.386	0.665
V_30_ (%)	5.2±8.5	4.0±6.1	4.0±6.2	4.0±6.2	0.126	0.142	0.047	0.625	0.112
V_40_ (%)	2.3±3.5	2.1±3.2	2.1±3.2	2.1±3.2	0.072	0.100	0.806	0.507	0.044
V_50_ (%)	1.3±1.9	1.4±2.1	1.2±1.8	1.2±1.8	0.790	0.013	0.514	0.502	0.011

P1: 1A vs. c-IMRT P2: 2A vs. c-IMRT P3: 2A vs.1A P4: 3A vs.2A P5: 3A vs. c-IMRT

### MUs and TTs

The delivery efficiency (in terms of the number of MUs and the TT) is shown in Table 4.

**Table 4:** Dosimetric results for number of monitoring units (MUs) and TTs ($\bar{X}\pm s$).

Variable	c-IMRT	1A	2A	3A	P1	P2	P3	P4	P5
MU	663±72	331±18	345±16	347±14	0.000	0.000	0.000	0.339	0.000
TT(sec)	189±15	62±1	149±1	239±1	0.000	0.000	0.000	0.000	0.000

P1: 1A vs. c-IMRT P2: 2A vs. c-IMRT P3: 2A vs.1A P4: 3A vs.2A P5: 3A vs. c-IMRT

The number of MUs for the 1A (331±18), 2A (345±16) and 3A (347±14) plans were reduced by 50%, 48% and 48%, respectively, over that of the c-IMRT plan (663±71). We calculated TT, and the result was IMRT(183±12 sec)、1A(68±4 sec)、2A(137±7 sec) and 3A(222±15 sec). All of the plans were also performed in the QA mode to record the TTs. Compared with the c-IMRT plan, there were substantial reductions of 67% and 21% in the TTs for the 1A and 2A plans (62±1 sec and 149±1 sec vs. 189±15 sec), whereas the TT of the 3A plan increased by 26% (239±1 sec).Plans 1A and 2A had a faster delivery than c-IMRT.

## Discussion

Brahme et al. developed a novel method in which the order of conventional treatment planning was reversed: the optimum incident beam dose distributions was determined from the desired dose distribution in the target volume in 1988[9]. Since then, c-IMRT has gradually become the preferred planning method for many cancers. A study has shown that the PTV coverage for a c-IMRT plan with 5F was comparable with those for 7F or 9F c-IMRT plans for upper thoracic EC. Fu et al.[10] found that the conformity of the c-IMRT plans did not improve further by increasing the number of fields beyond five. Wang et al.[11] reported that both 5- and 7F c-IMRT plans were optimal methods for upper thoracic EC. Therefore, we designed the c-IMRT plans with five fields in this study.

Over the past few years, it has been shown that VMAT, a novel form of IMRT, is dosimetrically superior to c-IMRT[5,12–13]. The most appropriate number of arcs used in the plans with respect to different disease sites remains a subject of debate. However, using more arcs in VMAT enables the superior modulation of factors during optimization by taking advantage of the independent optimization, the unrelated sequence of the MLC shape, the gantry speed and the dose rate combinations. Thus, increasing the number of arcs in a VMAT plan further optimizes the dose distribution in PTV in terms of conformity and homogeneity. However, VMATs with a larger number of arcs generally require more time for treatment delivery and quality assurance. Therefore, the appropriate arc number of VMATs remains to be determined.

The conformity and homogeneity of the dose distribution in the PTV are determined by the target volumes, the delivery equipment and the radiotherapy technology. Several studies have shown that a 1A VMAT plan may be dosimetrically less favorable than a fixed beam IMRT plan[14–16].

Our results showed that the 2A and 3A plans generated a superior CI for the PTV to the c-IMRT plan (0.88 and 0.89 vs. 0.86, P = 0.046,0.008), whereas there was no significant difference between the 1A VMAT and c-IMRT plans, except that the c-IMRT plan had a better HI than the 1A VMAT plan. Further analysis indicated that increasing the arc number of the VMAT plan improved the CI (0.88 and 0.89 for the 2A and 3A plans vs. 0.85 in the 1A plan) and the HI (1.07 and 1.06 in the 2A and 3A plans vs. 1.08 in the 1A plan) of the PTV. However, no significant difference was found between the 2A and 3A plans.

Significant advances have been made in radiotherapy delivery techniques over the past decades. These developments have been primarily driven by the need to reduce the radiation dose and the volume of normal tissues and organs, thereby minimizing the risks of toxicity and morbidity. Studies have shown that VMAT can deliver radiation treatment with equivalent or better OAR sparing than IMRT[8,17].

The spinal cord was given the highest priority for sparing among the OARs, and the D_max_ of the spinal cord has normally been limited to <45 Gy by NCCN guidelines. However, recently published reports have suggested a D_max_ for the spinal cord that is <40 Gy when RT is combined with chemotherapy[18–19]. This goal was met by the 2A, 3A and c-IMRT plans, whereas the D_max_ of the spinal cord in the 1A VMAT plan was slightly higher than 40 Gy: thus, the 1A VMAT plan may not be suitable for chemoradiotherapy of EC in terms of protecting the spinal cord.

The lung is another important OAR that should be considered during treatment planning. Our data showed that the dose constraints of the lungs were satisfied by all four plans. Moreover, compared to the c-IMRT plan, the 2A and 3A VMAT plans significantly reduced the lung volume that was irradiated with a moderate dose. Thus, the 2A and 3A VMAT plans have the potential to reduce the incidence of radiation pneumonitis. However, although the volume of lungs that was irradiated with a moderate dose (20 Gy or 30 Gy) was reduced using VMAT, a greater lung volume received a low dose (≤13 Gy) irradiation, which could compromise the prior advantage. It is unclear to what extent this consideration could affect the overall incidence of radiation pneumonitis. A study by Wang et al. [20–21] found that V_5_ was the only significant factor associated with treatment-related pneumonitis. A recent study conducted by MD Anderson showed that when the V_5_ of the lungs was limited to <65%, the risk of radiation pneumonitis was 24% for grade 2 and 12% for grade 3, and the related mortality rate was 1%. Thus, it was recommended that V_5_ be limited to within 65%[22]. In the current study, we observed only slight increases in V_5_ (1A 48.0, 2A 48.1, 3A 48.1 and 5F 47.3), V_10_ (1A 41.5, 2A 41.4, 3A 41.5 and 5F 39.6) and V_13_ (1A 35.9, 2A 35.3, 3A 35.5 and 5F 32.8) using the VMAT plans. More importantly, V_5_ in all of the plans was far below 65%. Thus, increasing low-dose irradiation of the lungs using VMAT should not compromise the advantage this plan offers in sparing OARs. For the heart, the reduction trend of heart parameters (V_30_, V_40_ and V_50_) was similar between 2A-VMAT and c-IMRT, and no difference in D_mean_ or V_20_ was found between VMAT and c-IMRT.

Our results also showed that the number of MUs required using the 1A (331±18), 2A (345±16) and 3A (347±14) plans were reduced by 50%, 48% and 48% compared to the number of MUs required for the c-IMRT plan (663±71), which was consistent with the results of a study by Clivio[20]. The significant increase in the number of MUs required in the IMRT plan has become a growing concern. An increase in the number of MUs may increase undesirable irradiation of normal tissues via the scattered dose, leading to an elevated risk of secondary cancers after treatment[21]. By contrast, the VMAT plan requires many fewer MUs because this plan is performed simultaneously with gantry rotation and a dynamic MLC adaptation to the target volume, instead of the sliding window technique that is used in c-IMRT. Thus, the application of VMAT may potentially reduce secondary cancer after radiation treatment.

Finally, VMAT also has a faster delivery than c-IMRT. In our study, the TTs for the 1A (62±1 sec) and 2A (149±1 sec) plans were reduced by 67% and 21%, respectively, compared to the c-IMRT plan (189±15 sec) although the TT of plan 3A (239±1sec) was increased by 26%. These results were consistent with those from a study on the fractional irradiation of malignant glioma in which the VMAT was 3.3 times as fast as IMRT[23]. Thus, the shorter treatment time and fewer MUs that are required for the VMAT plan compared with the c-IMRT plan could result in the VMAT plan becoming an important tool in image guided radiotherapy and hyper-fractionated accelerated radiotherapy[24].

A recent study[25]demonstrates that, using VMAT(two arcs)for patients with upper esophageal carcinoma, significantly reduces the delivery time and the dose to the lungs compared with IMRT. Our study’s conclusion on this point is similar. Furthermore, we compared VMAT plans with different number of arcs, and found that VMAT with two arcs is a good choice of radiotherapy techniques for upper esophageal carcinoma.

In conclusion, the 2A-VMAT plan appeared to spare the lungs from moderate-dose irradiation more than any of the other plans, at the expense of increasing the volume of low-dose irradiation, and also significantly reduced the number of required MUs and the TT over those of the c-IMRT plan. The dose conformity of the PTVs and OARs were improved when the arc number of the VMAT plan was increased from one to two; however, no further significant improvement was observed using the 3A-VMAT plan, except for an increased TT.
